# Synaptic interactions between perifornical lateral hypothalamic area, locus coeruleus nucleus and the oral pontine reticular nucleus are implicated in the stage succession during sleep-wakefulness cycle

**DOI:** 10.3389/fnins.2013.00216

**Published:** 2013-11-19

**Authors:** Silvia Tortorella, Margarita L. Rodrigo-Angulo, Angel Núñez, Miguel Garzón

**Affiliations:** Departamento de Anatomía, Histología y Neurociencia, Facultad de Medicina, Research Institute, Universidad Autonoma de Madrid, La Paz University Hospital (IDIPAZ)Madrid, Spain

**Keywords:** orexin, orexin-1 receptor, GABAergic receptors, catecholaminergic receptors, wakefulness, REM sleep

## Abstract

The perifornical area in the posterior lateral hypothalamus (PeFLH) has been implicated in several physiological functions including the sleep-wakefulness regulation. The PeFLH area contains several cell types including those expressing orexins (Orx; also known as hypocretins), mainly located in the PeF nucleus. The aim of the present study was to elucidate the synaptic interactions between Orx neurons located in the PeFLH area and different brainstem neurons involved in the generation of wakefulness and sleep stages such as the locus coeruleus (LC) nucleus (contributing to wakefulness) and the oral pontine reticular nucleus (PnO) nucleus (contributing to REM sleep). Anatomical data demonstrated the existence of a neuronal network involving the PeFLH area, LC, and the PnO nuclei that would control the sleep-wake cycle. Electrophysiological experiments indicated that PeFLH area had an excitatory effect on LC neurons. PeFLH stimulation increased the firing rate of LC neurons and induced an activation of the EEG. The excitatory effect evoked by PeFLH stimulation in LC neurons was blocked by the injection of the Orx-1 receptor antagonist SB-334867 into the LC. Similar electrical stimulation of the PeFLH area evoked an inhibition of PnO neurons by activation of GABAergic receptors because the effect was blocked by bicuculline application into the PnO. Our data also revealed that the LC and PnO nuclei exerted a feedback control on neuronal activity of PeFLH area. Electrical stimulation of LC facilitated firing activity of PeFLH neurons by activation of catecholaminergic receptors whereas PnO stimulation inhibited PeFLH neurons by activation of GABAergic receptors. In conclusion, Orx neurons of the PeFLH area seem to be an important organizer of the wakefulness and sleep stages in order to maintain a normal succession of stages during the sleep-wakefulness cycle.

## Introduction

Sleep and wakefulness are two mutually exclusive states that cycle with both ultradian and circadian periods. The brain mechanisms underlying the organization of the sleep-wakefulness cycle remain unclear. Many studies have identified several populations of neurons whose activity correlates with distinct behavioral states (Carter et al., 2012, 2013). The perifornical lateral hypothalamic area (PeFLH) has been implicated in several physiological functions including sleep-wakefulness regulation (McGinty and Szymusiak, 2003; Jones, 2008); this area contains a heterogeneous population of neuronal groups as reflected by both their state-dependent discharge properties and their neurotransmitter phenotypes. Among others, these cells express hypocretin/orexin (Orx), melanin-concentrating hormone, γ-aminobutyric acid (GABA) or glutamate (Vaughan et al., 1989; Bittencourt et al., 1992; de Lecea et al., 1998; Sakurai et al., 1998; Abrahamson and Moore, 2001; Rodrigo-Angulo et al., 2008). Orx neurons, mainly located in the perifornical (PeF) nucleus, have been extensively studied and implicated in the facilitation and/or maintenance of arousal (Alam et al., 2002; Koyama et al., 2003; Siegel, 2004; Sakurai, 2005; Takahashi et al., 2005; Sasaki et al., 2011); they are maximally active during active wakefulness and virtually cease firing during both slow wave sleep and rapid eye movement (REM) sleep (Lee et al., 2005a; Mileykovskiy et al., 2005).

Hypothalamic Orx projections make asymmetrical synaptic contacts with their target neurons in numerous brain areas implicated in the control of sleep-wakefulness cycle (Peyron et al., 1998; Horvath et al., 1999). Excitatory Orx effects have been demonstrated in neurons of arousal-related structures including the locus coeruleus (LC) nucleus (Horvath et al., 1999; Eggermann et al., 2001; Marcus et al., 2001; Burlet et al., 2002; Yamanaka et al., 2002; España et al., 2005; Cid-Pellitero and Garzon, 2011) where Orx promotes wakefulness (Bourgin et al., 2000; España et al., 2001; Thakkar et al., 2001; Xi et al., 2001). This effect may be due to an excitatory effect of Orx on LC neurons, as has been demonstrated *in vitro* (Hagan et al., 1999; Horvath et al., 1999; Ivanov and Aston-Jones, 2000; Soffin et al., 2002), suggesting that the effect of Orx on LC neurons may facilitate the arousal state.

Orx axon terminals and Orx receptors have also been identified in cholinoceptive areas of the pontine reticular formation involved in REM sleep generation (Greco and Shiromani, 2001; Marcus et al., 2001; Willie et al., 2003). Cholinoceptive neurons located in the ventral part of the oral pontine reticular nucleus (PnO) are involved in the generation and maintenance of REM sleep in rats (Horner and Kubin, 1999; Kohlmeier et al., 2002; Nuñez et al., 2002) and cats (Reinoso-Suárez et al., 1994, 1999, 2001; Garzón et al., 1998). Orx receptor subtypes are expressed in PnO neurons (Greco and Shiromani, 2001; Willie et al., 2003) and activation of such receptors enhances acetyl-choline release in the rat pons (Bernard et al., 2003, 2006). However, iontophoretic application of Orx in the PnO nucleus induced an inhibition of neuronal activity in anesthetized rats (Nuñez et al., 2006a); this effect was blocked by a previous iontophoretic application of bicuculline, indicating that inhibitory action of Orx involves the activation of GABA_A_ receptors.

The above mentioned results suggest that Orx neurons may play a crucial role in the organization of the sleep-wakefulness cycle and consequently, damage to Orx neurons could disrupt the normal succession of sleep-wake stages, as occurs in human narcolepsy and in animal models of this sleep disorder (Peyron et al., 2000; Thannickal et al., 2000). Narcolepsy is a neurological disorder characterized by severe daytime somnolence with a constellation of unusual symptoms that are best understood as intrusions of REM sleep phenomena into wakefulness (Guilleminault and Fromherz, 2005). In agreement with the hypothesis that Orx neurons play a role in narcolepsy these patients also have reduced cerebrospinal fluid levels of Orx (Nishino et al., 2000). Animal models exhibiting symptoms of narcolepsy show loss of Orx neurons or a decrease in Orx levels (Chemelli et al., 1999; Gerashchenko et al., 2001; Hara et al., 2001; Beuckmann et al., 2004). Canines with narcolepsy carry a mutation in the Orx-2 receptor (Lin et al., 1999) and mice with a deletion of this receptor (Willie et al., 2003) also show symptoms of narcolepsy.

The aim of the present study was to examine the synaptic interaction between Orx neurons located in the PeFLH area and different neuronal generators in the brainstem. We hypothesized that cross-talk among LC nucleus (contributing to wakefulness), PnO nucleus (contributing to REM sleep) and PeFLH area would orchestrate the sleep-wakefulness cycle. Anatomical and electrophysiological studies were undertaken to demonstrate this coordination of the sleep-wakefulness cycle.

## Materials and methods

Procedures were approved by the Ethics Board of the Universidad Autonoma de Madrid in accordance with the European Communities Council guidelines (2012/63/UE) on the ethical use of animals with every effort being made to minimize the suffering and number of animals employed. Fifty-five urethane-anesthetized (1.6 g/kg i.p.) Wistar rats (from Iffa-Credo, L'Arbresle, France) weighing 250–300 g were used for the physiological experiments. Animals were placed in a stereotaxic device with controlled body temperature (37°C). EEG activity was recorded through a macroelectrode stereotaxically placed into the frontal cortex [at 2 mm rostral to the Bregma and 2 mm from the midline (Paxinos and Watson, 2007)]. The EEG was filtered between 0.3 and 30 Hz, and amplified. Supplemental doses of the anesthetic were given when a decrease in the amplitude of the EEG delta waves was observed.

### Unit recording and drug application

Single-unit recordings were obtained with tungsten microelectrodes (2 MΩ) or glass micropipettes (World Precision Instruments, Sarasota, USA) filled with 2% neurobiotin in 0.5 M NaCl (Sigma) to locate the recording site. Three-barrel glass micropipettes were also used for unit recording and simultaneous application of pharmacological agents by microiontophoresis (see below).

Recording electrodes were placed at the LC nucleus (coordinates from Bregma: A, −9.3; L, 1.5 and depth, 7.0 mm), the PnO nucleus (coordinates from Bregma: A, −8.0; L, 1.0 and depth, 8.0 mm) or at the PeFLH area (coordinates from Bregma: A, −2.1; L, 1.0 and depth, 8.0 mm) by means of micromanipulators. Extracellular recordings were filtered (0.3–3 kHz), amplified (P15; GRASS Technology, Warwick, USA) and fed into a PC computer for off-line analysis by a Cambridge Electronic Design (CED; Cambridge, UK) 1401 interface at a sampling frequency of 10 kHz for the unit recordings, together with the EEG (sampling frequency of 200 Hz). Spike 2 software (CED) was used.

When barrel micropipettes were used, one of the glass capillaries was filled with a solution of NaCl (0.5 M) in order to record unit activity. A second micropipette was filled with bicuculline-methiodide (10 mM in 0.9% NaCl, pH 3.0, Sigma, St Louis, MO, USA). The remaining micropipette was filled with 0.5 M NaCl to balance the retention and ejection currents. Each barrel of the three-barreled pipette was connected via a silver wire to a channel on a microiontophoresis current generator (World Precision Instruments) that controlled retention and ejection currents for the drug-filled micropipette. Bicuculline was ejected with a positive current using a single 30-s pulse of 50 nA and negative retaining currents of 10–20 nA were used to delay drug leakage from the micropipette.

Bipolar stimulating electrodes (120 μm diameter blunt stainless steel wire) were placed at the ipsilateral PeFLH area, LC nucleus or in the PnO nucleus (same coordinates as described above). Electrical stimulation was performed using single rectangular pulses (0.1–0.3 ms, 50–100 μA) at 0.5 Hz or pulse trains of 500-ms duration at a frequency of 50 Hz, delivered through a GRASS S88 stimulator.

### Drugs

The hypocretin-1 receptor antagonist SB-334867 (100 μM; 100 nl) was applied through a 20G cannula connected to a Hamilton syringe (flow rate: 50 nl/min). The dose was similar to previous reports (Erami et al., 2012). Reserpine (methyl reserpate 3,4,5-trimethoxybenzoic acid ester, Sigma Chemical Co., St. Louis, USA) was dissolved in 50 μl of glacial acetic acid plus 0.9% NaCl (saline). The control solution consisted of saline plus 50 μl of glacial acetic acid in saline. Reserpine doses (1.0 and 5.0 mg/kg) were administered subcutaneously (s.c.) in a volume of 1.0 ml/kg of body weight. Unit recordings were performed 48 h after the reserpine injection.

### Electrophysiological data analysis

Analysis of the neocortical EEGs as well as the activity of each neuron was performed off-line in a PC computer. Spike 2 software was used to perform statistical calculations including summed peristimulus time histograms (PSTH), which were calculated using 2-ms bin widths and power spectra of the EEG. Statistical analyses were calculated with Student's two-tailed *t* tests for unpaired or paired data as required. Differences were considered statistically significant at a level of 95% (*p* < 0.05). All data are indicated as mean ± SEM.

### Anatomical studies

At the end of the electrophysiological experiments animals were injected i.p. with an overdose of pentobarbital and perfused transcardially with 4% paraformaldehyde in phosphate buffer pH 7.4. Brains were frozen, sectioned at 50 μm and collected in two series. In order to confirm the location of the recording electrodes, sections from the first series were processed to reveal the neurobiotin stained neurons: after rinsing in Tris saline buffer (TS) 0.1 M at pH 7.6, sections were incubated in a 1:150 Elite ABC kit (Vector) dilution in TS and 0.25% triton X-100 for 3 h to be developed with 0.05% 3-3′ DAB and 0.003% H_2_O_2_. Sections of the second series were processed for Nissl staining in order to corroborate the location of the stimulating electrodes.

For the anatomical connection tracing studies, retrograde Cholera Toxin-Alexia 594 (CT-A) and 1.5% Fluoro-Gold (FG) and anterograde FluoroRuby (FR) fluorescents tracers were injected in 16 adult rats of both sexes weighing 250–280 g. Under general anesthesia (a mixture of 50% ketamine, 40% atropine, and 10% valium i.p. 1 ml/250 g) animals were placed in the stereotaxic frame, and a craniotomy was made. Fluorescent injections were performed in PnO and LC nuclei, both in the same hemispheric side by means of a 1 μl Hamilton syringe (flow rate: 40 nl/min). In order to avoid contamination and tracer overlapping, injections in PnO nucleus were made from the contralateral side in an angle of 45° at coordinates from Bregma: A, −8.4; L, 1.5, and depth, 8.5, according to the (Paxinos and Watson, 2007); 300 nl of CT-A (10 animals), 40 nl of FG (3 animals), and 450 nl of FR (3 animals) were delivered in a single pulse. Injections in LC nucleus were performed with a vertical approach using a 1 μl Hamilton syringe stereotaxicaly aimed at the coordinates from Bregma: A, −9.6; L, 1.3; and depth 6.8; 50 nl of FG (12 animals), 50 nl of FR (2 animals), and 300 nl of CT-A (2 animals) were delivered. Animals were allowed to survive for 7 days before transcardial perfusion with 4% paraformaldehyde in 0.1 M phosphate buffer at pH 7.3 followed by increasing concentrations of sucrose solutions in the same buffer. Once removed, brains were stored in 30% sucrose solution during 5 days for cryopreservation and afterwards sectioned at 40 μm on a cryostat; sections were collected in three consecutive series devoted to fluorescence and Nissl staining. The third series was processed for Orx immunolabeling using the avidin-biotin-peroxidase method; sections were incubated with 1:1000 goat anti-Orx antiserum (Santa Cruz) in a solution containing 5% bovine serum albumin and 30% normal rabbit serum in Tris buffer 0.1 M at pH 7.6 for 48 h. After this, sections were incubated in 1:400 biotinylated rabbit anti-goat antibody solution (Chemicon) for 2 h and in 1:150 avidin-biotin-peroxidase reagent Elite ABC kit (Vector) for 1.5 h. The Orx immunoperoxidase was visualized by incubation in 0.05% 3-3′ DAB and 0.003% H_2_O_2_. Sections were studied under fluorescent or bright field illumination using a Zeiss Axioskop microscope.

## Results

### Anatomical results

**Figure 1:**
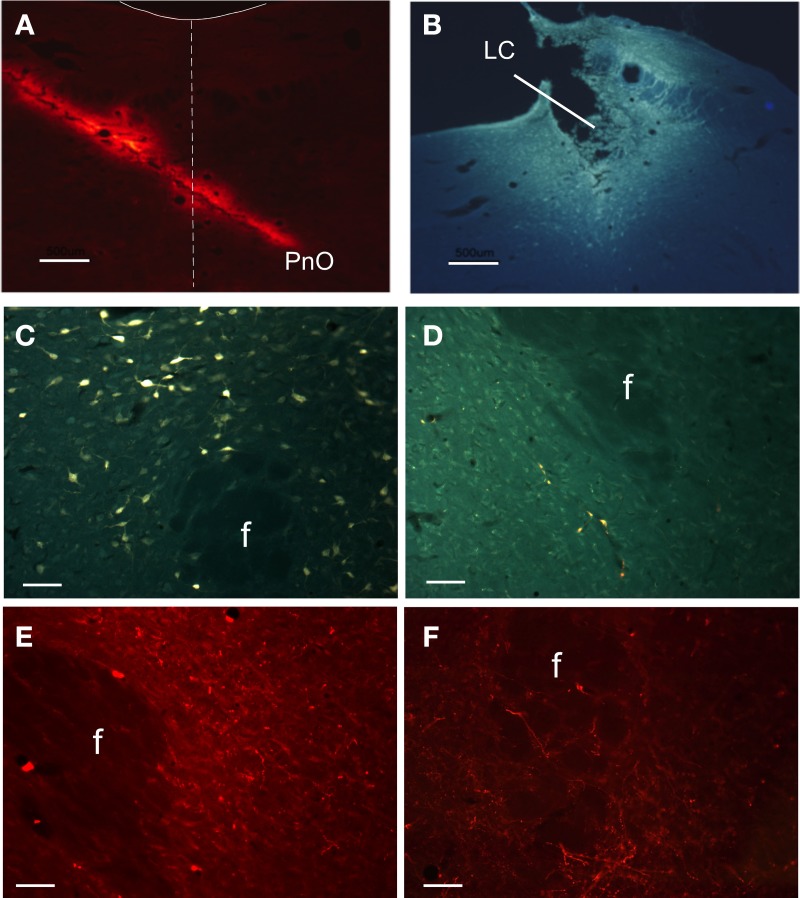
**Anatomical projections between PeFLH area and LC or PnO nuclei**. **(A)** Microphotograph showing a representative case of FR injection aimed at PnO from the contralateral side. Dash line indicates midline; solid line indicates the IV ventricle contour. **(B)** Microphotograph of a FG injection aimed at LC. **(C,D)** Microphotographs showing labeled neurons in the PeF area after FG injection in PnO nucleus **(C)** and LC nucleus **(D)**. **(E,F)** Microphotographs of two cases showing fiber terminals after FR injections in PnO nucleus **(E)** and LC nucleus **(F)**. f: fornix. Calibration bars in **(A)** and **(B)**, 500 μm. Calibration bars in **(C–F)**, 150 μm.

To determine the projections from the PeF area, two retrograde tracers CT-A and FG were injected into the LC and PnO nuclei, respectively (Figures 1A,B). Also, the anterograde FR fluorescent tracer was used. Numerous retrograde labeled neurons were observed in several hypothalamic structures after PnO tracer injections, as we have already reported (España et al., 2005; Nuñez et al., 2006a; Figure 1C). The injections of the anterograde tracer in PnO nucleus labeled fibers and terminals only in the lateral sector of PeFLH area (Figure 1E). Neurons labeled after retrograde tracer injections in LC nucleus were located in the ventral sector of PeFLH area and intermingled with the fibers and terminals that could be observed after anterograde tracer injections in LC nucleus (Figures 1D,F). In all cases, the presence of double-labeled neurons was very low (<0.5% of labeled neurons).

**Figure 2:**
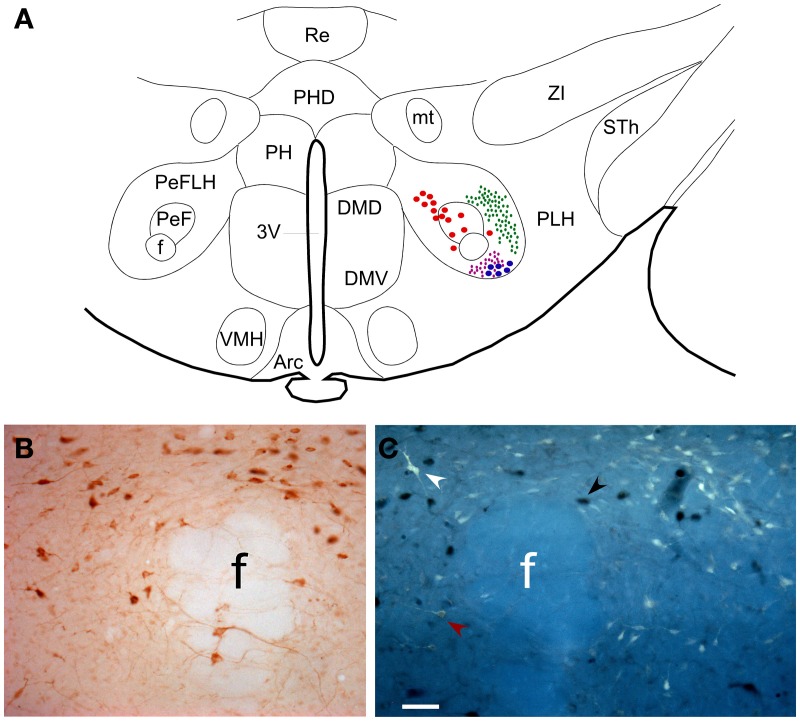
**(A)** Schematic drawing of the hypothalamic area in a coronal section of rat brain at antero-posterior coordinates Bregma −3.36 mm. It is shown the distribution of neurons projecting to PnO (red dots) and LC (blue dots) and the distribution of fiber terminals arising PnO and LC are represented in green and purple small dots, respectively. **(B)** Microphotograph of PeF showing neurons immunocytochemically stained for anti-Orx. **(C)** Microphotograph showing adjacent section showed in **(B)** where Orx neurons are pointed by black arrowheads, FG neurons projecting to LC by white arrowheads and double labeled neurons by red arrowheads. Calibration bar: 50 μm. Abbreviations: 3V: 3rd ventricle; Arc: arcuate hypothalamic nucleus; DMD: dorsomedial hypothalamic nucleus, dorsal part; DMV: dorsomedial hypothalamic nucleus, ventral part; f: fornix; mt: mammillothalamic tract; PeF: perifornical nucleus; PeFLH: perifornical part of lateral hypothalamus area; PH: posterior hypothalamic nucleus; PHD: posterior hypothalamic area, dorsal part; PLH: peduncular part of lateral hypothalamus; Re: reuniens thalamic nucleus; Sth: subthalamic nucleus; VMH: ventromedial hypothalamic nucleus; ZI: zona incerta.

A schematic drawing of the hypothalamic area depicting differential distribution of retrograde labeled neurons and anterograde labeled fibers after PnO and LC tracer injections is shown in Figure 2A. As has been previously reported (Nuñez et al., 2006a) Orx immunoreactive neurons were clustered to the PeF nucleus (Figure 2B). In the PeF nucleus we found double-labeled neurons projecting to PnO (data not shown) or to LC nucleus (Figure 2C) that were also stained for Orx.

### PeF activation of LC

**Figure 3:**
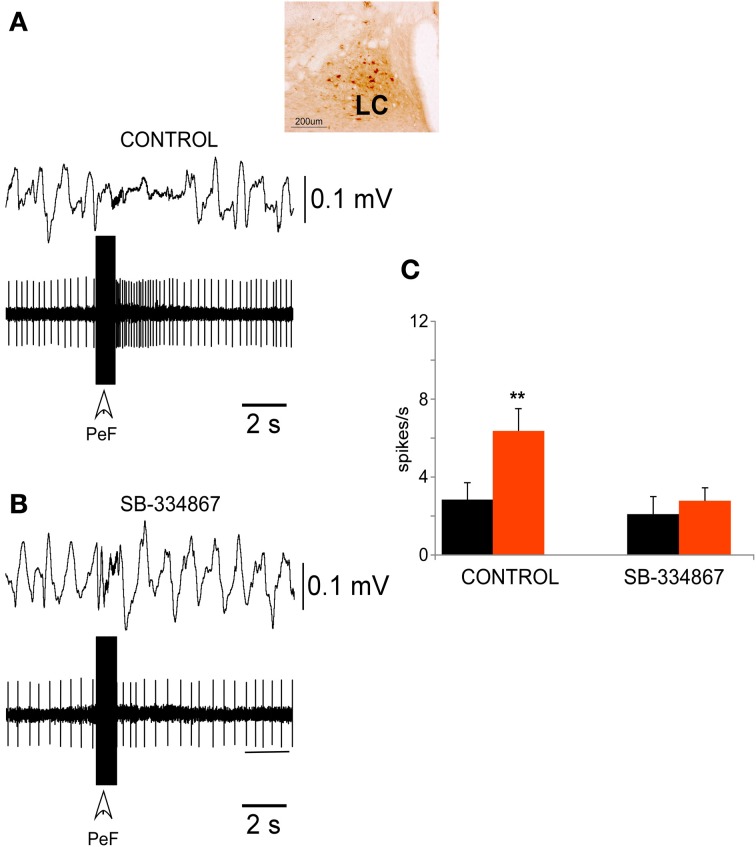
**PeFLH electrical stimulation excited LC neurons**. **(A)** A train of electrical stimuli (50 Hz; 50 μA; 500 ms of duration) in the PeFLH area (vertical arrow) evoked a long-lasting increase in LC neuronal activity and a decrease of the EEG slow waves. The inset shows the recording area, which was stained with neurobiotin. **(B)** This effect was blocked by the injection of the Orx-1 receptor antagonist SB-334867 into the LC by means of a Hamilton syringe (100 μ M; 100 nl). **(C)** Plot of the mean neuronal activity measured during a 10 s period before (black) and after (red) the stimulus train in the PeFLH area. In control conditions (*n* = 12 cells) LC neuronal activity increased, but the effect was blocked by the Orx-1 receptor antagonist. The asterisk indicates significant statistical differences (^**^p < 0.01).

The LC neurons displayed a non-rhythmic discharge pattern with a mean spontaneous frequency of 3.4 ± 1.0 spikes/s (*n* = 40). The recording site was established by iontophoretic injection of neurobiotin through the recording micropipette (Figure 3, inset).

Trains of electrical stimulus delivered in the PeFLH area (50 Hz; 50 μA; 500 ms of duration) increased the mean firing rate of LC neurons from 3.4 ± 1.0 to 9.8 ± 1.7 spikes/s, measured 10 s after the stimulation train (*p* < 0.001; Figures 3A,C). The excitatory effect was observed in 64% of LC neurons (32 out of 50 cells), and could last up to 30 s. The EEG pattern changed from continuous slow waves evoked by the anesthetic to a faster activity evoked by PeFLH stimulation (Figure 3A). In spontaneous conditions the percentage of delta waves (1–4 Hz) in the EEG was 98.6 ± 1.8% and the percentage of faster (>4 Hz) waves was 1.4 ± 0.9%. During the period of 10 s after the PeFLH stimulation train the proportion of delta waves decreased to 67.3 ± 3.4% and the percentage of >4 Hz waves increased to 32.7 ± 4.6% (*p* < 0.001), indicating that the PeFLH stimulation induced an activation of the EEG.

The excitatory effect evoked by PeFLH stimulation in LC neurons was blocked by the injection of the Orx-1 receptor antagonist SB-334867 into the LC nucleus. The SB-334867 was applied by means of a Hamilton syringe (100 μ M; 100 nl) and the effect was tested 15 min. after application when the drug concentration was stabilized. Trains of electrical stimulus delivered in the PeFLH area (50 Hz; 50 μA; 500 ms of duration) increased the mean firing rate (calculated during 10 s before and 10 s after the stimulation train) of LC neurons from 2.8 ± 0.8 to 6.3 ± 1.2 spikes/s, (*n* = 12; *p* < 0.001; Figures 3A,C). The excitatory responses evoked by PeFLH stimulation train in LC neurons were blocked by the SB-334867 (Figure 3B). Trains of electrical stimulus delivered in the PeFLH area did not increase the mean firing rate of LC neurons from 2.1 ± 0.9 to 2.8 ± 0.6 spikes/s (*n* = 12; *p* = 0.12; Figures 3B,C).

### PeF inhibition of PnO activity

**Figure 4:**
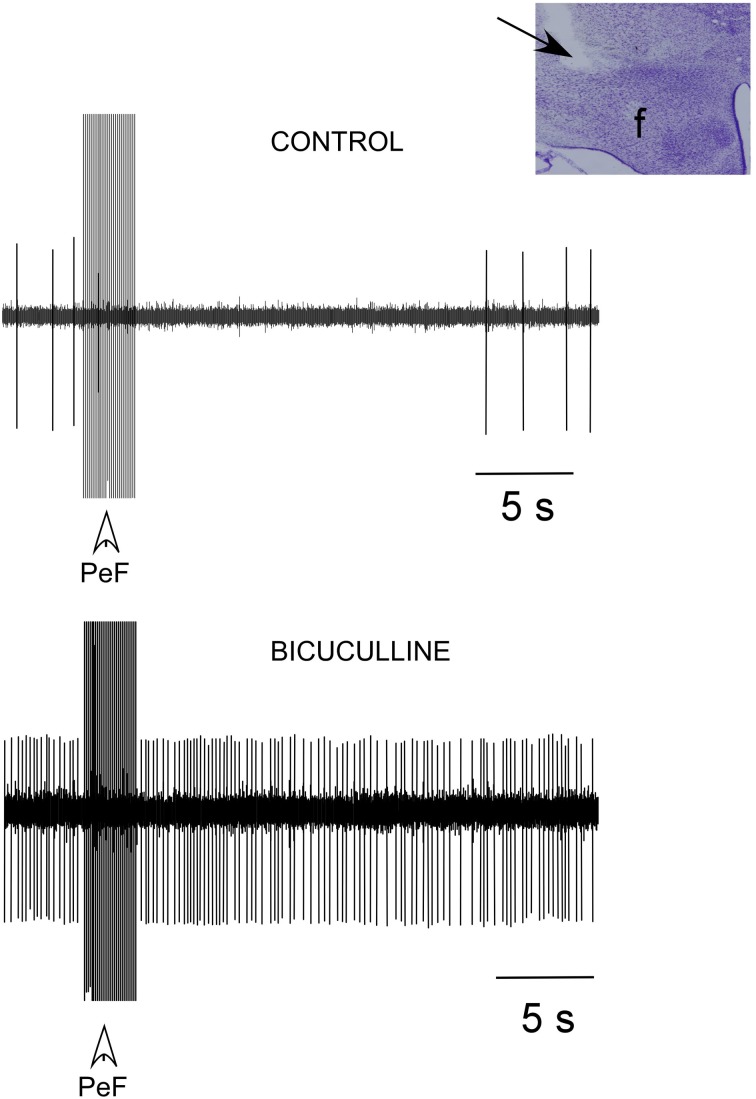
**PeFLH stimulation inhibited PnO neuronal activity**. A train of electrical stimuli (50 Hz; 50 μA; 500 ms of duration) in the PeFLH area (vertical arrow) evoked a long-lasting inhibition of PnO neurons (upper trace). The inhibitory effect was blocked by the iontophoretic application of the GABA_A_ receptor antagonist bicuculline (10 mM) into the PnO nucleus (lower trace). Inset shows the stimulated PeFLH area in the Nissl stained section (arrow).

Most PnO neurons (15 out of 18 neurons; 83%) decreased their spontaneous firing rate from 1.2 ± 0.3 Hz in control conditions to 0.2 ± 0.4 Hz (*p* < 0.001) 10 s after application of a train of electrical stimuli delivered in the PeFLH area (50 Hz; 50 μA; 500 ms of duration; Figure 4, upper trace). To determine if the inhibitory response evoked by PeF stimulation was due to activation of GABAergic receptors, bicuculline (a GABA_A_ receptor antagonist; 10 mM) was iontophoretically applied (*n* = 8). Bicuculline ejection increased the firing rate of PnO neurons to 4.9 ± 0.9 Hz. In the presence of bicuculline, successive PeFLH stimulation trains did not change the firing rate of PnO neurons (Figure 4, lower trace), indicating that the inhibitory effect of PeF stimulation was due to activation of GABAergic receptors within the PnO nucleus, in agreement with previous reports (Nuñez et al., 2006a).

### PeF neuronal responses to LC or PnO stimulation

Unit recordings were performed in the PeFLH area (*n* = 39). Neurons displayed a non-rhythmic discharge pattern with a mean spontaneous frequency of 1.3 ± 0.3 spikes/s. PeFLH neurons were identified by their stereotaxic coordinates after reconstruction of the electrode track.

**Figure 5:**
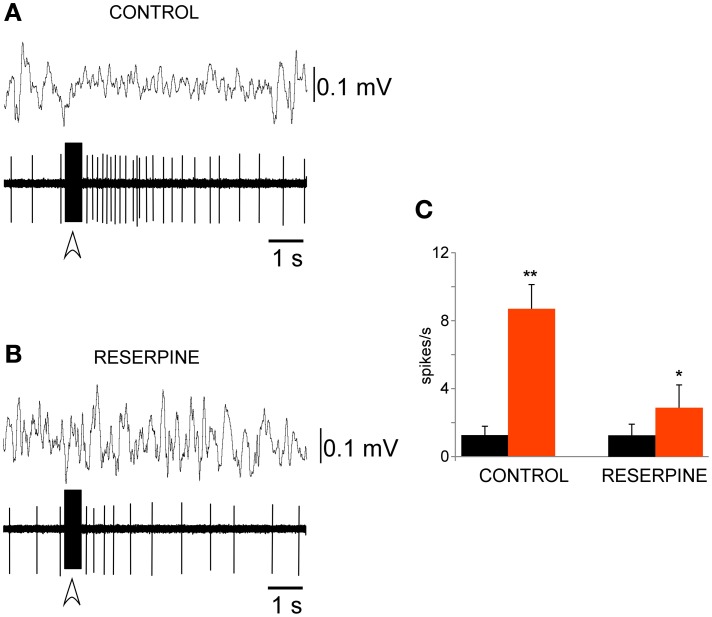
**LC stimulation evoked a long–lasting excitation of PeFLH neurons**. **(A)** Train stimulation of the LC evoked an increase of PeF neuronal activity for 3 s, with a simultaneous decrease in EEG slow waves. The stimulation train is indicated by the open arrow. **(B)** LC-evoked excitation was abolished in a reserpine-treated animal. **(C)** Plot of the mean firing rate calculated for the 10 s period before (black) and after (red) LC stimulation train in control animals (*n* = 21 cells) and in reserpine-treated animals (*n* = 12 cells). The LC-evoked excitation of PeFLH neurons was reduced after catecholaminergic depletion. The asterisk indicates significant statistical differences (^*^p < 0.05; ^**^p < 0.01).

The PeFLH area is implicated in the control of the sleep-wakefulness cycle, facilitating wakefulness (see Introduction). We study the effect on PeFLH neuronal activity of electrical stimulation of brainstem areas implicated in the generation of wakefulness (LC nucleus) or REM sleep (PnO nucleus). Trains of electrical stimuli delivered in the LC nucleus (50 Hz; 50–100 μA; 500 ms of duration) induced a 1–10 s increase in firing rate, in most PeFLH neurons (21 out of 23 cells; 91%; Figure 5A). The mean firing rate increased from 1.3 ± 0.3 spikes/s in spontaneous conditions to 8.6 ± 1.4 spikes/s (*n* = 21; *p* < 0.001; Figure 5C). The percentage of delta waves decreased during the same time period from 96.5 ± 4.1 to 79.8 ± 2.9% (*p* < 0.001).

The alkaloid reserpine which depletes catecholamines from nerve endings have been used. Reserpine administration (1 mg/kg, once daily for three consecutive days) reduced the response of the PeFLH neurons to LC train stimulation. The LC-evoked rise in the firing rate was reduced in the presence of reserpine. Under reserpine activity rose from 1.3 ± 0.5 spikes/s before the stimulation train to 2.8 ± 1.4 spikes/s after the stimulation train; *n* = 8; *p* = 0.015; Figures 5B,C, indicating that the LC-evoked excitation of PeF neurons was mediated by the activation of catecholaminergic fibers.

**Figure 6:**
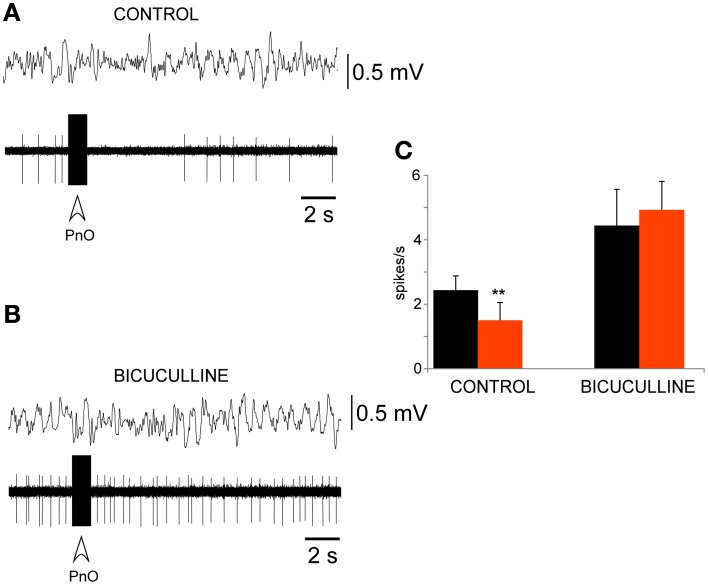
**PnO electrical stimulation evoked a long-lasting inhibition of PeFLH neurons**. **(A)** Electrical train stimulation of the PnO nucleus evoked a decrease in PeFLH neuronal activity for 5 s accompanied by a slight decrease in EEG slow waves. The stimulation train is indicated with open arrow. **(B)** The PnO-evoked inhibition was abolished by bicuculline (10 mM). **(C)** Plot of the mean firing rate calculated for the 10 s period before (black) and after (red) the PnO stimulation train in control animals (*n* = 14 cells) and 5 min after bicuculline application (*n* = 8 cells). The PnO-evoked inhibition was blocked by the GABA_A_ receptor antagonist. The asterisk indicates significant statistical differences (^**^p < 0.01).

To determine if there is a reciprocal connection between the PeFLH area and PnO nucleus, electrical stimulation of the PnO nucleus (0.5 Hz; 50–100 μA; 0.1–0.3 ms of duration) was applied during unit recordings of PeFLH neurons. Trains of electrical stimulus delivered in the PnO nucleus (50 Hz; 50–100 μA; 500 ms of duration) induced a decrease of the firing rate in 14 out of 17 PeFLH neurons during 1–8 s (82%; Figure 6A). The remaining neurons did not display significant changes in their firing rate. The mean firing rate of these neurons affected by PnO train stimulation was 2.4 ± 0.5 spikes/s in spontaneous conditions and this decreased to 1.4 ± 0.6 spikes/s during 2–5 s after the stimulation train (*n* = 14; *p* = 0.009). PnO train stimulation induced a short-lasting decrease of slow waves, probably by activation of the basal forebrain through polysynaptic pathways (Camacho Evangelista and Reinoso Suarez, 1964; Nuñez et al., 2006b, Teruel-Marti et al., 2008).

The inhibition of PeFLH neuronal activity by PnO electrical stimulation was blocked by iontophoretic application of bicuculline (10 mM; *n* = 8; Figure 6B). Figure 5C shows the mean firing rate in control conditions (*n* = 14) and under bicuculline (10 mM; *n* = 8). The spontaneous firing rate of PeFLH neurons increased to 4.4 ± 1.2 spikes/s 5 min after bicuculline application and, in these conditions, PnO stimulation did not alter the firing rate of the neurons (5.0 ± 0.9 spikes/s), indicating that the PnO-evoked inhibition of PeFLH neurons was dependent on the GABA_A_ receptor activation.

## Discussion

The present results demonstrate using anatomical and electrophysiological methods the existence of a neuronal network involving the PeFLH area, LC, and PnO nuclei that would control the sleep-wake cycle. The Orx PeF neurons favor EEG activation by excitatory projections to the LC nucleus and simultaneously block REM sleep generation by inhibition of PnO neurons through GABAergic receptors. This study also reveals that the LC and PnO nuclei exerted a feedback control on neuronal activity of PeFLH area in order to maintain a normal succession of stages during the sleep-wakefulness cycle. Top-down and bottom-up regulatory mechanisms are engaged to control the succession of sleep-wakefulness stages.

Both Orx-1 and Orx-2 receptors are expressed in the rodent brainstem (Greco and Shiromani, 2001; Marcus et al., 2001). Whereas the distribution of both receptors is quite similar in the PnO nucleus (Greco and Shiromani, 2001; Cluderay et al., 2002; Brischoux et al., 2008), LC nucleus shows a much more prominent expression for Orx-1 receptor mRNA and protein (Trivedi et al., 1998; Greco and Shiromani, 2001); Orx-2 receptors appear to be virtually absent in noradrenergic neurons of the LC nucleus (Brischoux et al., 2008). In agreement with these results, our data show that application of the Orx-1 receptor antagonist SB-334867 into the LC nucleus prevents the excitatory effect evoked by PeFLH stimulation. The lesion of neurons expressing the Orx-2 receptor in the lateral hypothalamus, by using the toxin Orx 2-saporin, have already revealed the implication of this receptor in different narcoleptic signs (Gerashchenko et al., 2001, 2003). The additional utilization of selective Orx-2 antagonists in the future could further help elucidate differential roles in regions expressing both receptors, such as the PnO.

The PeFLH area has been implicated in the regulation of behavioral arousal during wakefulness (Kilduff and Peyron, 2000; Siegel, 2004; Szymusiak and McGinty, 2008). This area contains Orx neurons mainly located in the PeF nucleus that are active during wakefulness and silent or with a low activity during non-REM and REM sleep (Alam et al., 2002; Koyama et al., 2003; Lee et al., 2005a, b; Suntsova et al., 2007). In a previous study we showed that Orx PeF neurons activate GABAergic receptors to inhibit PnO neurons, controlling the onset of REM sleep and thus, facilitating wakefulness (Nuñez et al., 2006a). In agreement with this observation, Lu and collaborators demonstrated that activation of PeF cells with bicuculline blocked the ability of pontine carbachol injections to elicit REM sleep (Lu et al., 2007). In the present study we show that there is a reciprocal connection between PnO and PeF nuclei. Both pathways have an inhibitory effect mediated by the activation of GABA_A_ receptors because effects were blocked by local application of the GABA_A_ receptor antagonist bicuculline. This experiment does not discard that other pathways could activated by PeFLH stimulation through other neurotransmitter actions. Consequently, when PeF neurons are active they block the possibility of REM sleep generation while PnO neurons activation during REM sleep inhibits PeF neurons and prevents wakefulness. Moreover, GABAergic sleep-active anterior hypothalamic neurons project to the PeF nucleus and GABA release is increased in this region during slow wave sleep (Nitz and Siegel, 1996; Saper et al., 2001, 2005; Uschakov et al., 2006). Thus, during slow wave and REM sleep PeF Orx neurons are inhibited. The absence of Orx is associated with narcolepsy, a disorder manifested by an uncontrollable occurrence of REM sleep (Chemelli et al., 1999; Lin et al., 1999; Thannickal et al., 2000). In agreement with this, Orx knock-out mice show an increase of slow wave and REM sleep during the darkness period, whereas wakefulness is decreased (Chemelli et al., 1999).

In this study we further demonstrated *in vivo* that PeF neurons facilitate wakefulness by direct excitation of LC neurons, which contribute to arousal by excitation of thalamic and cortical neurons (McCormick and Prince, 1988; McCormick, 1989; Aston-Jones, 2005). Previous studies in brain slices or neuronal cultures have demonstrated that Orx depolarizes LC neurons, increasing their firing rate (Soffin et al., 2002; Murai and Akaike, 2005). Also, the firing rates of LC neurons increased after microiontophoretic injection of Orx (Bourgin et al., 2000). LC neurons recorded in the present study showed similar spontaneous firing rate in control conditions from anesthetized rats that previous studies (Aston-Jones and Bloom, 1981; Bourgin et al., 2000) and also are consistent with these previous studies in demonstrating that Orx increases neuronal excitability in the LC nucleus through the activation of the Orx-1 receptor, since excitability is diminished by the Orx-1 receptor antagonist SB-334867 (Gompf and Aston-Jones, 2008). Orx innervation of LC neurons projecting to the cortex has been reported recently (Cid-Pellitero and Garzon, 2011). This Orx innervation probably supplies excitatory inputs to LC nucleus that are critical for cortical activation in transitions from sleep to wakefulness and during EEG activation.

The activity of LC neurons is involved in maintenance of wakefulness and EEG activation. These neurons are active during wakefulness, decrease their firing rate during slow wave sleep and are silent during REM sleep (Hobson and McCarley, 1975; Aston-Jones and Bloom, 1981). Unilateral lesions of the LC nucleus in cats enhance REM sleep (Caballero and De Andres, 1986). Carter and collaborators, using an optogenetic approach to stimulate or inhibit LC neurons found that silencing LC neurons blocked Orx-mediated sleep-to-wakefulness transitions while increasing the excitability of LC neurons enhanced these transitions (Carter et al., 2012, 2013).

Consequently with the above results, the LC nucleus plays a key role in regulating the sleep-wakefulness cycle. To favor the wakefulness stage, LC neurons activate PeF neurons by a direct pathway from LC nucleus to PeFLH area, as shown here. Moreover, electrical stimulation of LC nucleus induced an increase in the neuronal firing rate of PeF neurons by activation of catecholaminergic receptors. The lateral hypothalamus receives a moderately dense noradrenergic innervation (Baldo et al., 2003), most of which arises from outside the LC nucleus (Yoshida et al., 2006). Our study shows that neurons labeled after retrograde tracer injections in LC nucleus were located in the ventral sector of PeFLH area. Previous studies have described dense projections from PeFLH to the LC in the rat (Luppi et al., 1995; España et al., 2005; Lee et al., 2005b) and in the cat (Torterolo et al., 2013). The PeFLH neurons projecting to the LC distribute within the hypothalamic region containing the Orx cell group without any observed rostrocaudal or mediolateral topography, as occur in our experiments. However, retrogradely-labeled lateral hypothalamic neurons after LC nucleus tracer injections have been reported to locate also in the dorsal half of the Orx-containing cell group (España et al., 2005). This difference may be due to the tracer infusion volume or the tracer uptake.

It is reasonable to believe that both effects, inhibition of REM sleep generation and facilitation of arousal, could be performed by the same PeF neuronal population. However, our anatomical results indicate there are two different neuronal populations sending separate projections to PnO and LC nuclei. In fact, injections of retrograde tracers in PnO nucleus resulted in labeled neurons in the PeF nucleus and in the medial sector of the PeFLH area, while neurons labeled after retrograde tracer injections in LC nucleus were located in the ventral sector of PeFLH area. Taken together, these results suggest the existence of a neuronal network between the lateral hypothalamus and brainstem structures that may control the appropriate succession of the stages during sleep-wakefulness cycle. Moreover, Orx neurons of the PeFLH area seem to be an important organizer of the wakefulness and sleep stages based on their anatomical projections and synaptic interactions with different brainstem “sleep generators.” Thus, some sleep disorders such as narcolepsy or insomnia may be due to alterations of the Orx system.

## Author contributions

Conceived and designed the experiments: Angel Núñez, Miguel Garzón, and Margarita L. Rodrigo-Angulo. Performed and analyzed electrophysiological experiments: Angel Núñez and Silvia Tortorella. Performed and analyzed anatomical experiments: Miguel Garzón and Margarita L. Rodrigo-Angulo. Wrote the paper: Angel Núñez, Miguel Garzón, and Margarita L. Rodrigo-Angulo.

### Conflict of interest statement

The authors declare that the research was conducted in the absence of any commercial or financial relationships that could be construed as a potential conflict of interest.
